# Construction of Taiwanese Adult Reference Phantoms for Internal Dose Evaluation

**DOI:** 10.1371/journal.pone.0162359

**Published:** 2016-09-12

**Authors:** Shu-Jun Chang, Shih-Yen Hung, Yan-Lin Liu, Shiang-Huei Jiang

**Affiliations:** 1 Institute of Nuclear Engineering and Science, National Tsing Hua University, Hsinchu, Taiwan; 2 Health Physics Division, Institute of Nuclear Energy Research, Atomic Energy Council, Taoyuan, Taiwan; 3 Chi Mei Medical Center, Liouying, Tainan, Taiwan; 4 Department of Medical Imaging and Radiological Science, Central Taiwan University of Science and Technology, Taichung, Taiwan; University of North Carolina at Chapel Hill, UNITED STATES

## Abstract

In the internal dose evaluation, the specific absorbed fraction (SAF) and S-value are calculated from the reference phantom based on Caucasian data. The differences in height and weight between Caucasian and Asian may lead to inaccurate dose estimation. In this study, we developed the Taiwanese reference phantoms. 40 volunteers were recruited. Magnetic resonance images (MRI) were obtained, and the contours of 15 organs were drawn. The Taiwanese reference man (TRM) and Taiwanese reference woman (TRW) were constructed. For the SAF calculation, the differences in the self-absorption SAF (self-SAF) between the TRM, TRW, and Oak Ridge National Laboratory (ORNL) adult phantom were less than 10% when the difference in organ mass was less than 20%. The average SAF from liver to pancreas of TRM was 38% larger than that of the ORNL adult phantom, and the result of TRW was 2.02 times higher than that of the ORNL adult phantom. For the S-value calculation, the ratios of TRW and ORNL adult phantom ranged from 0.91 to 1.57, and the ratios of TRM and ORNL adult phantom ranged from 1.04 to 2.29. The SAF and S-value results were dominantly affected by the height, weight, organ mass, and geometric relationship between organs. By using the TRM and TRW, the accuracy of internal dose evaluation can be increased for radiation protection and nuclear medicine.

## Introduction

The importance of internal dose evaluation increases with the use of radiopharmaceuticals and the demand for radiation protection. Radioisotopes that enter the human body accumulate in various organs. In addition to receiving self-absorbed doses, tissues and organs are also exposed to the radionuclides accumulated in other organs. Therefore, the retention of radioactive substances, the volume and shape of tissues and organs, and the geometric relationship between organs should be considered for internal dose evaluation.

The American Society of Nuclear Medicine published the Medical Internal Radiation Dose (MIRD) methodology to assess internal doses for various radionuclides and organs [1,2]. The S-value from a source organ to a target organ is multiplied by the cumulative activity of the source organ to obtain the average absorbed dose of the target organ. Family anthropomorphic phantoms developed by the Oak Ridge National Laboratory (ORNL) are a commonly employed set of mathematical models for S-value simulation [3–6]. The organ models are described primarily using quadric mathematical equations; for example, a cylindroid for the torso and an ellipsoid for the stomach. These phantoms are categorized as MIRD-type stylized phantoms.

The MIRD-type phantoms are mainly established using the statistics of Caucasians [7,8], without considering the differences in height and weight of various ethnicities. Asians account for over 50% of the world’s population. The International Atomic Energy Agency (IAEA) highlighted that the average organ volume of Asians is markedly less than that of Caucasians [9]. Moreover, differences in the organ mass may exist among populations from different regions in Asia because of their diverse dietary habits, lifestyles, and geographical environments. Therefore, region-specific reference phantoms should be constructed for radiation protection of internal exposure.

Regarding the construction of Asian reference man, the Korean reference man, which possessed a height of 170.2 cm and a weight of 68.2 kg, was established [10,11]. Kim et al. [12] calculated the specific absorbed fractions (SAFs) of the Korean reference man. The results indicated that when the difference in height and weight was not significant, the mass of organs was the major factor influencing the internal dose. Yamauchi et al. [13] built a mathematical phantom with a height and weight of 170 cm and 65 kg based on Japanese statistical data. The Chinese reference phantom was also created, and the photon SAF was calculated accordingly [14]. However, the SAFs from the Chinese phantom are not directly comparable to those from other phantoms.

As an island country, Taiwan’s geographical features and climate conditions, as well as lifestyles and dietary habits of the local people, differ markedly from those of the countries mentioned previously. The objective of this study was to construct Taiwanese reference phantoms. The Monte Carlo technique was employed to simulate the SAF and S-value. The accuracy of internal dose evaluation for radiation protection and nuclear medicine can be improved for Taiwanese.

## Materials and Methods

### Organ mass for Taiwanese adults

This study was approved by the Human Experiment and Ethics Committee, Central Taiwan University of Science and Technology (File no: 002). All participants signed an informed consent. Based on a national survey of the body type and obesity among people residing in Taiwan [15], the average height and weight of adults older than 19 years old were taken as the external dimensions to construct the reference phantoms for Taiwanese male and female. The statistics for male were 168.7 cm in height and 69.0 kg in weight, and those for female were 156.2 cm in height and 56.6 kg in weight. 40 volunteers, including 20 males and 20 females, whose height and weight were within ±3% differences of the average values were recruited with an average age of 33 ranging from 20 to 65 years.

The participants underwent 1.5T MRI (Sonata, Siemens Medical System, Germany) with the fast spin-echo (FSE) pulse sequence to produce T2-weighted images. In FSE, the 180°-refocusing pulse was applied, leading to less susceptibility artefact. Subsequently, the edge of organs in T2-weighted images is more pronounced than the edge in T1-weighted images. The head, neck, thorax, abdomen, and pelvis were scanned separately with pixel sizes of 0.9×1.0, 0.8×0.9, 1.4×1.8, 1.4×1.6, and 1.2×1.4 mm, respectively. The slice thickness was 4 mm for head and neck, 5 mm for thorax and abdomen, and 6 mm for pelvis. Three radiologists with over two years of MRI experience were invited to draw the contours of the brain, eyes, thyroid, lungs, heart (wall), liver, stomach (wall), spleen, gall bladder, pancreas, kidneys, urinary bladder (wall), ovaries, uterus, and testicles. The length of the head and neck, torso, and legs as well as the lateral distance and anterior-posterior distance of the head and torso were also measured. The organ volume was calculated by multiplying the voxel number in the contour by the voxel size; the organ mass was the product of the organ volume and organ density. The tissue density was primarily employed from the IAEA-TECDOC-1005 report [9].

### Construction of Taiwanese reference phantoms

The ORNL adult phantom (178.6 cm in height and 73.7 kg in weight) was constructed based on the mathematical parameters of the family phantom series [6]. The length of the head and neck, torso, and legs in the z direction of the ORNL adult phantom was then scaled to the average length of the head and neck, torso, and legs of the Taiwanese, respectively. The modified male phantom had a head and neck length of 27.0 cm, a torso of 64.7 cm, and legs of 77.0 cm, whereas the female phantom had a head and neck of 25.2 cm, a torso of 58.1 cm, and legs of 72.9 cm. The equations for the head, trunk, and legs are listed below. The parameters for the x and y axes of the Taiwanese reference man (TRM) and Taiwanese reference woman (TRW) were adopted from the average values measured using the MR images as shown in Table 1.

Head:

$${\left(\frac{x}{A_{H}}\right)}^{2}+{\left(\frac{y}{B_{H}}\right)}^{2}\leq 1,\;\text{and}\;C_{T}\leq z\leq C_{H}$$ (1)

Trunk:

$${\left(\frac{x}{A_{T}}\right)}^{2}+{\left(\frac{y}{B_{T}}\right)}^{2}\leq 1,\;\text{and}\;0\leq z\leq C_{T}$$ (2)

Legs:

$${\left(x\right)}^{2}+{\left(y\right)}^{2}\leq \pm \left(A_{T}+\frac{A_{T}}{C_{L}}Z\right),\;\text{and}\;-C_{L}\leq z\leq 0$$ (3)

**Table 1:** Mathematical parameters for the head, trunk, and leg models in the TRM, TRW, and ORNL adult phantom.

Phantom	*A*_*H*_	*B*_*H*_	*C*_*H*_	*A*_*T*_	*B*_*T*_	*C*_*T*_	*C*_*L*_
ORNL adult phantom	8.0	10.0	28.6	20.0	10.0	70.0	80.0
TRM	7.8	9.7	27.0	18.0	9.7	64.7	77.0
TRW	7.6	9.4	25.2	16.9	9.4	58.1	72.9

15 organs were redesigned based on the formulas of the ORNL adult phantom [6]. The coefficients of equations in the z axis were adjusted according to the body height ratio between the Taiwanese reference phantom and the ORNL adult phantom. The coefficients in the x and y axes were modified to meet the average organ volume calculated from the MRI results, while the ratio of the coefficients was maintained as used in the ORNL adult phantom. The elemental compositions of organs and tissues were employed from the ORNL TM-2007 report [16]. Table 2 lists the elemental compositions and densities of organs and tissues for the Taiwanese reference phantoms.

**Table 2:** Elemental compositions and densities of organs and tissues for the Taiwanese reference phantoms.

Organ	Density (g/cm^3^)	H	C	N	O	Ca	Na	P	S	Cl	K
Brain	1.03	10.7	14.5	2.2	71.2	-	0.2	0.4	0.2	0.3	0.3
Eyes	1.03	9.6	19.5	5.7	64.6	-	0.1	0.1	0.3	0.1	-
Thyroid	1.05	10.4	11.9	2.4	74.5	-	0.2	0.1	0.1	0.2	0.1
Breasts	1.02	11.6	51.9	-	36.5	-	-	-	-	-	-
Lungs	0.26	10.3	10.5	3.1	74.9	-	0.2	0.2	0.3	0.3	0.2
Heart	1.03	10.4	13.9	2.9	71.8	-	0.1	0.2	0.2	0.2	0.3
Stomach	1.05	10.6	11.5	2.2	75.1	-	0.1	0.1	0.1	0.2	0.1
Liver	1.06	10.3	18.6	2.8	67.1	-	0.2	0.2	0.3	0.2	0.3
Kidneys	1.05	10.3	13.2	3.0	72.4	0.1	0.2	0.2	0.2	0.2	0.2
Pancreas	1.05	10.6	16.9	2.2	69.4	-	0.2	0.3	0.1	0.2	0.2
Spleen	1.06	10.3	11.3	3.2	74.1	-	0.1	0.3	0.2	0.2	0.3
Small intestine	1.04	10.6	11.5	2.2	75.1	-	0.1	0.1	0.1	0.2	0.1
Large intestine	1.04	10.6	11.5	2.2	75.1	-	0.1	0.1	0.1	0.2	0.1
Urinary bladder	1.04	10.5	9.6	2.6	76.1	-	0.2	0.2	0.2	0.3	0.3
Testes	1.04	10.6	9.9	2.0	76.6	-	0.2	0.1	0.2	0.2	0.2
Ovaries	1.05	10.5	9.3	2.4	76.8	-	0.2	0.2	0.2	0.2	0.2
Uterus	1.04	10.6	31.5	2.4	54.7	-	0.1	0.2	0.2	0.1	0.2
Skin	1.1	10	20.4	4.2	64.5	-	0.2	0.1	0.2	0.3	0.1
Bone	1.4	7.3	25.5	3.1	47.9	10.2	0.3	5.1	0.2	0.1	0.1
Muscle	1.05	10.2	14.3	3.4	71.0	-	0.1	0.2	0.3	0.1	0.4
Soft tissue	1.03	10.5	25.6	2.7	60.2	-	0.1	0.2	0.3	0.2	0.2

### Monte Carlo simulation

The Monte Carlo N-Particle Transport Code (MCNP version 5) [17] was used to construct the geometric models of TRM and TRW. The SAFs of target organs from mono-energetic photons emitted from various source organs were simulated. Additionally, ^131^I and ^99m^Tc were used as source terms and S-values from various source organs to target organs were simulated for internal dose evaluation in nuclear medicine. The radioactive decay data were adopted from the Data of the Radiation Dose Assessment Resource (RADAR) [18]. The SAF and S-value results of the TRM, TRW, ORNL adult phantom, ORNL female phantom, Japanese reference phantom, Korean reference phantom, and OLINDA/EXM [19] were compared.

An eight-node parallel computing cluster system, consisting of an quad-core Intel Xeon E5606 CPU (2.13 GHz) and 3,940-MB memory in each node, was used to perform Monte Carlo simulation [20–22]. In each case, energy deposition from ten million particles was tallied to ensure most of the simulation results with the coefficient of variation (CV) under 10%. CV, defined as the ratio of the standard deviation σ to the mean μ, is a standardized measurement of dispersion. Due to the assumption that the source is uniformly distributed in the source organ and the radiation is isotropically emitted, 10% CV is generally reliable for the internal dose evaluation. When the CV exceeded 20%, the simulation result was excluded.

## Results and Discussion

**Fig 1:**
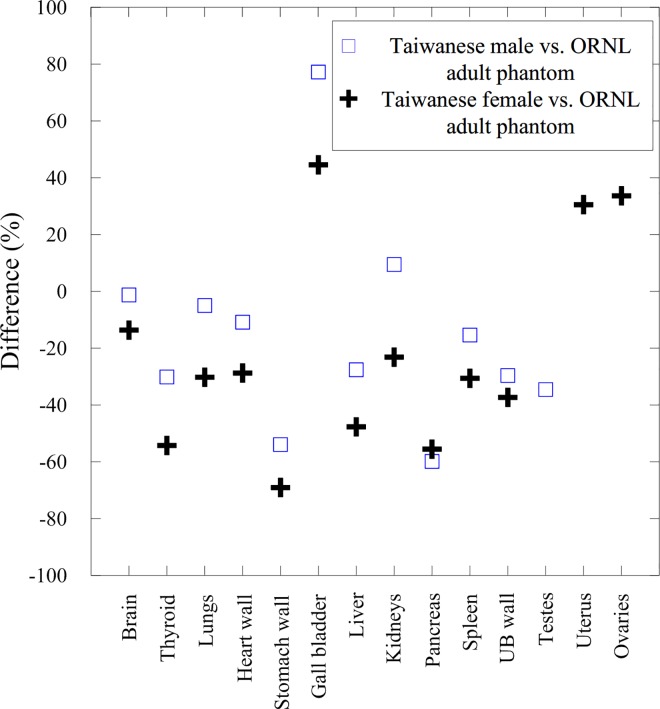
Comparison of the average organ mass between Taiwanese male vs. ORNL adult phantom and Taiwanese female vs. ORNL adult phantom.

**Fig 2:**
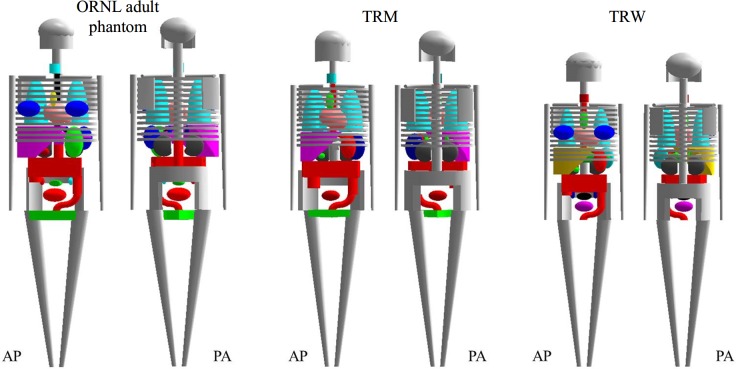
3D models of the ORNL adult phantom, TRM, and TRW in the anterior-posterior (AP) view and posterior-anterior (PA) view. The skin and muscle have been removed to reveal the skeletal system and inner organs.

Table 3 lists the average organ volume of Taiwanese adults and the ORNL adult phantom. Fig 1 depicts the percentage differences in the organ mass between the Taiwanese population and ORNL adult phantom. For the Taiwanese female, the organ masses were all lower than those of the ORNL adult phantom except the gall bladder, uterus, and ovaries. For the Taiwanese male, the differences for most organs were less than 40%; only three organs exhibited differences of more than 50%, including the gall bladder, stomach wall, and pancreas. The main explanation is that the participants underwent the MRI scan were fasted for at least four hours, resulting in gall bladder expansion and stomach contraction. Additionally, the partial volume effect of MRI may be responsible for the under-estimated wall volume of the hollow organs. Fig 2 shows the 3D models of the ORNL adult phantom, TRM, and TRW; the skin and muscle were removed to reveal organs and the skeletal system.

**Table 3:** Average organ volume of Taiwanese male, female, and the ORNL adult phantom.

	Taiwanese male	Taiwanese female	ORNL adult phantom
Brain	1353	1183	1370
Thyroid	13.9	9.14	19.9
Lungs	3212	2358	3380
Heart wall	270	216	303
Heart content	390	311	437
Stomach wall	74.2	47.0	152
Stomach content	122	77.3	250
Liver	1325	957	1830
Gall bladder	17.9	14.6	10.1
Kidneys	315	221	288
Pancreas	36.4	40.3	90.7
Spleen	149	122	176
UB wall	20.0	18.9	45.7
UB content	85.0	84.0	203
Testes	24.6	-	37.6
Uterus	-	99.2	76.0
Ovaries	-	11.2	8.38
Bone	5808	5094	7221

**Fig 3:**
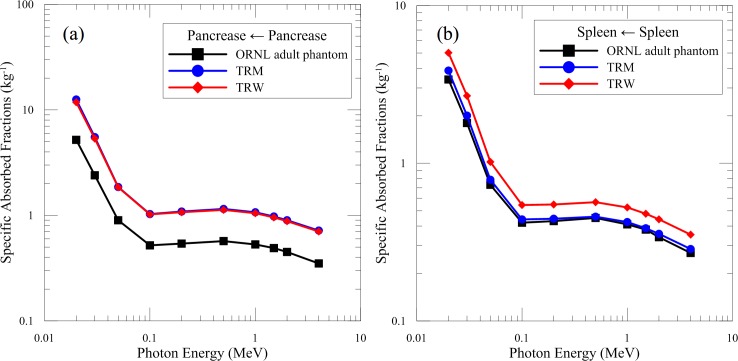
Self-SAFs of the TRM, TRW, and ORNL adult phantom. (a) Pancreas and (b) spleen were simulated.

When the source organ and target organ are the same, SAF denotes as the self-SAF. Fig 3 shows the self-SAFs of the pancreas and spleen as a function of photon energy. The average self-SAFs of the pancreas calculated from TRM and TRW were approximately two times larger than the result calculated from the ORNL adult phantom. The significant discrepancy is primarily because the organ mass of the Taiwanese reference phantoms is 50% smaller than that of the ORNL adult phantom. Concerning other organs with the mass difference less than 20%, the differences in the self-SAF were all less than 10%, such as the spleen of TRM as shown in Fig 3B.

Table 4 lists the self-SAFs of the liver, pancreas, and spleen from the ORNL adult phantom, TRM, and TRW at 0.03, 0.1, and 1 MeV. The data from the ORNL female phantom, Japanese reference phantom [23], and Korean reference phantom [12] were also compared. The self-SAFs of TRM for the liver and spleen differed from those of the Korean phantom by less than 8%. For the pancreas, the average result of TRM was 19% higher than those of the Korean phantom. The average self-SAFs of TRW for the liver and spleen were 45% and 17% higher than those of the ORNL female phantom, respectively, whereas the average difference increased to 87% for the pancreas. This is mainly because the lower organ mass and the difference in organ geometry.

**Table 4:** Self-SAFs (kg^-1^) of the liver, pancreas, and spleen from the ORNL adult phantom, ORNL female phantom, Japanese reference phantom, Korean reference phantom, TRM, and TRW at 0.03, 0.1, and 1 MeV.

Energy (MeV)	ORNL adult	ORNL female	Japanese	Korean	TRM	TRW
	Liver					
0.03	0.280	0.364	0.430	0.400	0.399	0.562
0.1	0.093	0.111	0.120	0.130	0.119	0.156
1	0.081	0.098	0.110	0.100	0.104	0.136
	Pancreas					
0.03	2.400	2.660	2.400	4.260	5.520	5.350
0.1	0.520	0.563	0.510	0.910	1.030	1.020
1	0.530	0.591	0.480	0.930	1.070	1.050
	Spleen					
0.03	1.800	2.130	3.400	2.070	2.010	2.680
0.1	0.420	0.476	0.680	0.460	0.440	0.544
1	0.410	0.478	0.680	0.450	0.424	0.525

**Fig 4:**
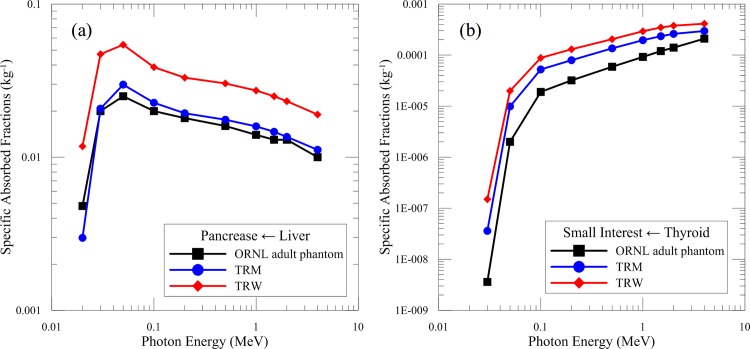
SAFs of the TRM, TRW, and ORNL adult phantom. (a) SAF(pancreas←liver) and (b) SAF(small interest←thyroid) were simulated.

When the source organ and the target organ are not the same, the SAF denotes as SAF(target←source). Fig 4A shows the SAF(pancreas←liver), where the average difference between TRM and the ORNL adult phantom was less than 38%. The average SAF of TRW was 2.02 times higher than that of the ORNL adult phantom. This is primarily because the torso of TRW is thinner than that of the ORNL adult phantom; therefore, the liver and pancreas are located closer together. Regarding the SAF(small intestine←thyroid) as shown in Fig 4B, the results calculated from TRM and TRW exceeded those of the ORNL adult phantom by 3.31 and 8.28 times, respectively. This is mainly because TRM and TRW were 5.5% and 12.5% shorter than the ORNL adult phantom, respectively, resulting in shorter distance between organs and thus higher absorbed fractions.

Table 5 shows the SAFs from liver to pancreas, spleen, and kidneys for various reference phantoms at 0.03, 0.1, and 1 MeV. For the SAF(pancreas←liver) and SAF(kidneys←liver), the results of TRM were mostly between those of the ORNL adult phantom and the ORNL female phantom. For the SAF(spleen←liver), the average SAF of TRM was 20% larger than the result of the ORNL female phantom. Most of the SAFs of TRW were larger than those of other phantoms. Again, this is because the height of TRW was the shortest among phantoms. The distance between organs was relatively close.

**Table 5:** SAFs (kg^-1^) from liver to pancreas, spleen, and kidneys for the ORNL adult phantom, ORNL female phantom, Korean reference phantom, TRM, and TRW at 0.03, 0.1, and 1 MeV.

Energy (MeV)	ORNL adult	ORNL female	Korean	TRM	TRW
	Pancreas←liver				
0.03	2.02×10^−2^	2.93×10^−2^	2.31×10^−2^	2.08×10^−2^	2.71×10^−2^
0.1	1.99×10^−2^	2.58×10^−2^	3.00×10^−2^	2.27×10^−2^	3.87×10^−2^
1	1.43×10^−2^	1.87×10^−2^	2.18×10^−2^	1.59×10^−2^	2.72×10^−2^
	Spleen←liver				
0.03	5.40×10^−4^	1.14×10^−3^	3.95×10^−4^	1.47×10^−3^	1.93×10^−3^
0.1	3.60×10^−3^	5.60×10^−3^	3.71×10^−3^	6.69×10^−3^	8.31×10^−3^
1	3.40×10^−3^	4.85×10^−3^	3.06×10^−3^	5.44×10^−3^	6.49×10^−3^
	Kidneys←liver				
0.03	1.40×10^−2^	1.85×10^−2^	1.61×10^−2^	1.66×10^−2^	2.79×10^−2^
0.1	1.50×10^−2^	1.80×10^−2^	2.11×10^−2^	1.81×10^−2^	2.56×10^−2^
1	1.20×10^−2^	1.42×10^−2^	1.53×10^−2^	1.39×10^−2^	1.91×10^−2^

**Fig 5:**
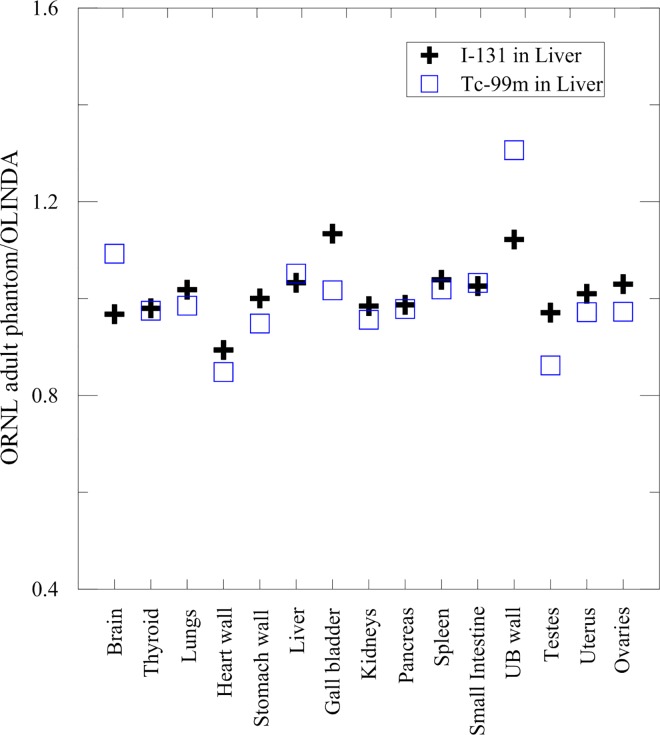
S-value ratios between the ORNL adult phantom and the OLINDA/EXM. The liver was the source organ, and ^131^I and ^99m^Tc were the source terms, respectively.

Regarding the internal dose evaluation of radioisotopes using the MIRD schema, the S-values of TRM, TRW, and ORNL adult phantom were simulated and compared to the S-values provided by OLINDA/EXM. Fig 5 shows the S-value ratios of the ORNL adult phantom and OLINDA/EXM with the liver as the source organ and ^99m^Tc and ^131^I as the radioisotope, respectively. Most S-value ratios were between 0.9 to 1.1, which verifies the geometric modeling and the Monte Carlo simulation of the ORNL adult phantom.

Table 6 lists the S-values from the kidneys, liver, lungs, and thyroid calculated based on TRM with ^131^I in the source organ. The CVs for the self-absorbed S-values were less than 0.2%, and the CVs of the remaining S-values were mostly within 5% except the S-value from the thyroid to the testes and urinary bladder. This is because the significant distance between the source and target organs and the small volume of the target organ prevent the convergence of the simulation. Table 7 lists the S-values from the kidneys, liver, lungs, and thyroid of TRM with ^99m^Tc in the source organ. The CVs for the self-absorbed S-values were all within 0.1%. Similarly, when the source organ was the thyroid and the target organs were the testes and urinary bladder wall, the S-values had a CV larger than 20% and cannot converge. Tables 8 and 9 present the S-values and %CV for TRW. Besides the instances that involved the long distance between source/target organs and the small target volume, the statistical errors of Monte Carlo simulation were less than 5%.

**Table 6:** S-values (mGy/MBq.s) and %CV of TRM with ^131^I in the kidneys, liver, lungs, and thyroid, respectively.

Target organ	Kidneys	Liver	Lungs	Thyroid
Brain	2.69E-09 (4.0%)	6.06E-09 (2.6%)	3.02E-08 (1.2%)	2.41E-07 (0.4%)
Thyroid	1.91E-08 (11%)	4.56E-08 (7.5%)	3.26E-07 (3.1%)	2.17E-03 (0.2%)
Lungs	2.70E-07 (0.4%)	7.32E-07 (0.3%)	4.82E-05 (0.1%)	3.39E-07 (0.4%)
Heart wall	2.40E-07 (0.6%)	7.39E-07 (0.3%)	1.47E-06 (0.2%)	2.11E-07 (0.6%)
Stomach wall	7.29E-07 (1.2%)	6.36E-07 (1.0%)	4.32E-07 (1.0%)	2.94E-08 (4.1%)
Liver	9.11E-07 (0.3%)	3.08E-05 (0.1%)	6.95E-07 (0.4%)	4.84E-08 (0.9%)
Kidneys	1.08E-04 (0.1%)	9.41E-07 (0.4%)	2.68E-07 (0.7%)	2.10E-08 (2.4%)
Pancreas	1.57E-06 (1.1%)	1.09E-06 (1.5%)	3.91E-07 (1.6%)	2.82E-08 (5.6%)
Spleen	2.65E-06 (0.8%)	3.56E-07 (0.9%)	4.91E-07 (0.6%)	3.67E-08 (2.7%)
Small Intestine	6.31E-07 (0.3%)	5.39E-07 (0.3%)	8.23E-08 (0.8%)	7.17E-09 (3.4%)
LLI wall	3.28E-07 (1.2%)	1.30E-07 (1.5%)	4.13E-08 (2.4%)	4.09E-09 (8.8%)
ULI wall	6.72E-07 (0.6%)	5.63E-07 (0.6%)	8.47E-08 (1.6%)	6.93E-09 (5.0%)
UB wall	9.78E-08 (3.6%)	6.47E-08 (4.2%)	1.26E-08 (9.3%)	-
Testes	2.50E-08 (7.8%)	1.92E-08 (9.4%)	4.07E-09 (18%)	-

**Table 7:** S-values (mGy/MBq.s) and %CV of TRM with ^99m^Tc in the kidneys, liver, lungs, and thyroid, respectively.

Target organ	Kidneys	Liver	Lungs	Thyroid
Brain	3.61E-10 (5.9%)	9.74E-10 (3.5%)	6.68E-09 (1.3%)	6.97E-08 (0.4%)
Thyroid	4.59E-09 (13%)	1.26E-08 (7.3%)	1.03E-07 (2.6%)	1.99E-04 (0.1%)
Lungs	8.89E-08 (0.4%)	2.60E-07 (0.3%)	4.84E-06 (0.1%)	1.13E-07 (0.4%)
Heart wall	8.10E-08 (0.5%)	2.67E-07 (0.3%)	5.27E-07 (0.2%)	6.84E-08 (0.6%)
Stomach wall	2.67E-07 (0.6%)	2.29E-07 (0.8%)	1.51E-07 (0.8%)	7.76E-09 (4.3%)
Liver	3.28E-07 (0.3%)	4.28E-06 (0.1%)	2.47E-07 (0.4%)	1.30E-08 (1.0%)
Kidneys	1.25E-05 (0.1%)	3.35E-07 (0.4%)	8.77E-08 (0.7%)	4.82E-09 (2.8%)
Pancreas	5.65E-07 (0.6%)	4.03E-07 (0.9%)	1.40E-07 (1.3%)	6.80E-09 (6.4%)
Spleen	9.32E-07 (0.2%)	1.27E-07 (0.8%)	1.74E-07 (0.5%)	9.38E-09 (2.8%)
Small Intestine	2.28E-07 (0.3%)	1.91E-07 (0.3%)	2.37E-08 (0.8%)	1.39E-09 (3.2%)
LLI wall	1.07E-07 (0.8%)	3.95E-08 (1.5%)	1.00E-08 (2.6%)	5.98E-10 (11%)
ULI wall	2.45E-07 (0.5%)	2.03E-07 (0.6%)	2.49E-08 (1.6%)	1.40E-09 (6.5%)
UB wall	2.87E-08 (3.5%)	1.74E-08 (4.4%)	2.71E-09 (12%)	-
Testes	5.66E-09 (9.0%)	3.59E-09 (11%)	-	-

**Table 8:** S-values (mGy/MBq.s) and %CV of TRW with ^131^I in the kidneys, liver, lungs, and thyroid, respectively.

Target organ	Kidneys	Liver	Lungs	Thyroid
Brain	4.28E-09 (3.2%)	9.28E-09 (2.3%)	4.74E-08 (1.0%)	2.75E-07 (0.4%)
Thyroid	3.08E-08 (11%)	6.27E-08 (8.2%)	4.30E-07 (3.3%)	3.28E-03 (0.2%)
Breasts	9.26E-08 (1.3%)	2.76E-07 (0.8%)	6.35E-07 (0.5%)	1.48E-07 (1.1%)
Lungs	3.18E-07 (0.6%)	8.13E-07 (0.4%)	7.01E-05 (0.2%)	3.99E-07 (0.5%)
Heart wall	3.13E-07 (0.5%)	9.17E-07 (0.3%)	1.85E-06 (0.2%)	2.64E-07 (0.6%)
Stomach wall	8.20E-07 (0.8%)	6.89E-07 (1.0%)	4.50E-07 (1.1%)	3.50E-08 (4.2%)
Liver	1.31E-06 (0.2%)	4.26E-05 (0.1%)	9.18E-07 (0.2%)	6.93E-08 (0.9%)
Kidneys	1.51E-04 (0.1%)	1.29E-06 (0.4%)	3.60E-07 (0.8%)	3.10E-08 (2.4%)
Pancreas	2.10E-06 (0.7%)	1.89E-06 (0.8%)	7.96E-07 (1.1%)	5.66E-08 (4.0%)
Spleen	2.77E-06 (0.3%)	4.28E-07 (0.9%)	7.66E-07 (0.6%)	5.56E-08 (2.4%)
Small Intestine	1.04E-06 (0.3%)	6.48E-07 (0.3%)	1.17E-07 (0.7%)	1.09E-08 (2.3%)
LLI wall	1.45E-07 (1.4%)	6.27E-08 (2.3%)	1.95E-08 (4.0%)	2.05E-09 (13%)
ULI wall	5.61E-07 (0.8%)	5.41E-07 (0.7%)	7.88E-08 (2.0%)	7.40E-09 (5.6%)
UB wall	9.85E-08 (3.2%)	7.03E-08 (3.7%)	1.52E-08 (7.7%)	-
Ovaries	3.82E-07 (3.0%)	2.18E-07 (3.8%)	4.84E-08 (8.1%)	-
Uterus	3.38E-07 (1.1%)	2.08E-07 (1.4%)	4.47E-08 (3.0%)	4.16E-09 (9.5%)

**Table 9:** S-values (mGy/MBq.s) and %CV of TRW with 99mTc in the kidneys, liver, lungs, and thyroid, respectively.

Target organ	Kidneys	Liver	Lungs	Thyroid
Brain	6.21E-10 (4.5%)	1.59E-09 (2.9%)	1.13E-08 (1.1%)	7.84E-08 (0.4%)
Thyroid	6.47E-09 (13%)	1.94E-08 (7.1%)	1.37E-07 (2.8%)	2.96E-04 (0.2%)
Breasts	2.31E-08 (1.3%)	8.03E-08 (0.7%)	1.93E-07 (0.5%)	3.68E-08 (1.0%)
Lungs	1.08E-07 (0.5%)	2.91E-07 (0.3%)	6.81E-06 (0.1%)	1.31E-07 (0.4%)
Heart wall	1.08E-07 (0.5%)	3.36E-07 (0.3%)	6.64E-07 (0.2%)	8.42E-08 (0.6%)
Stomach wall	3.03E-07 (0.6%)	2.53E-07 (0.9%)	1.57E-07 (0.9%)	1.03E-08 (4.3%)
Liver	4.71E-07 (0.2%)	5.75E-06 (0.1%)	3.28E-07 (0.2%)	1.94E-08 (0.9%)
Kidneys	1.70E-05 (0.1%)	4.67E-07 (0.4%)	1.22E-07 (0.7%)	7.18E-09 (2.7%)
Pancreas	7.71E-07 (0.5%)	6.95E-07 (0.6%)	2.92E-07 (0.9%)	1.60E-08 (3.9%)
Spleen	9.69E-07 (0.2%)	1.55E-07 (0.8%)	2.73E-07 (0.5%)	1.58E-08 (2.5%)
Small Intestine	3.77E-07 (0.2%)	2.34E-07 (0.3%)	3.52E-08 (0.7%)	2.14E-09 (2.9%)
LLI wall	4.71E-08 (1.3%)	1.81E-08 (2.3%)	4.84E-09 (4.2%)	2.37E-10 (18%)
ULI wall	2.05E-07 (0.6%)	1.94E-07 (0.6%)	2.40E-08 (1.9%)	1.63E-09 (6.9%)
UB wall	3.06E-08 (3.0%)	1.94E-08 (3.7%)	2.83E-09 (9.6%)	-
Ovaries	1.32E-07 (2.6%)	7.30E-08 (3.5%)	1.23E-08 (8.3%)	-
Uterus	1.13E-07 (1.0%)	6.80E-08 (1.3%)	1.04E-08 (3.1%)	7.17E-10 (11%)

**Fig 6:**
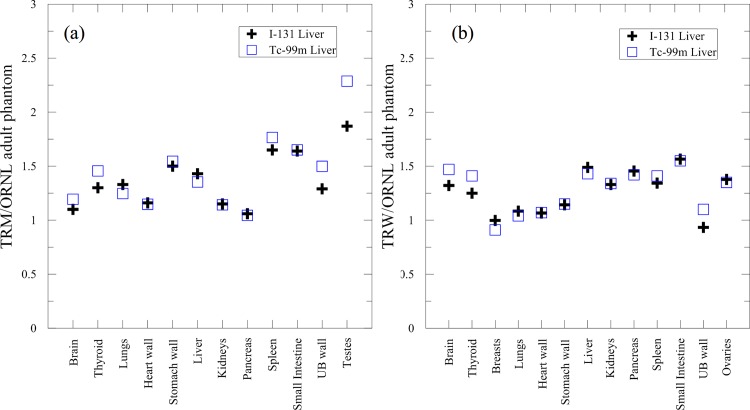
**S-value ratios of (a) TRM/ORNL adult phantom and (b) TRW/ORNL adult phantom.** The liver was the source organ, and ^131^I and ^99m^Tc were the source terms, respectively.

Fig 6 shows the S-value ratios of TRM/ORNL adult phantom and TRW/ORNL adult phantom when the source organ was the liver. The ratios of TRM/ORNL adult phantom ranged between 1.04 and 2.29, whereas the ratios of TRW/ORNL adult phantom ranged between 0.91 and 1.57. The reason that most of the S-value ratios exceeded one is that the distance between the source and target organs is shorter than that of the ORNL adult phantom. In addition, the mass of the target organ is smaller than that of the ORNL adult phantom.

The differences in the tissue and organ density between the Taiwanese reference phantoms and the ORNL phantoms were mostly less than 2%, except the eyes and breasts. However, these two tissues are less important in internal dose evaluation. In the calculation of SAF and S-value, SAF is defined as the fraction of energy emitted from the source region that is deposited in the target region per mass of the target region. The increased density increases the organ mass, but also increases the energy absorbed coefficient. Therefore, the difference in density between phantoms may not markedly affect the SAF and S-value results.

The S-values of TRM and TRW differed markedly from those of the ORNL adult phantom. This is because of their differences in the height, weight, organ mass, and geometric relationship. Clairand et al. [24] highlighted that when using an anthropomorphic phantom with a 20-cm difference in height, the S-value ratio can reach 4.6. In this study, the SAF results calculated from TRM and TRW also shows 3.31 and 8.28 times higher than those derived from ORNL adult phantom. The use of the ORNL adult phantom typically leads to underestimation of the internal dose and the corresponding risk from internal exposure. The Taiwanese reference phantoms can be used for radiation protection and nuclear medicine to increase the accuracy of internal dose evaluation. The limitation of this study is that some S-value results do not converge due to the long distance between the source and target organs. The traditional reciprocity method and buildup factor method can be applied based on the organ models to calculate the S-value and SAF. In the future, the Taiwanese reference phantoms can also be applied to simulate the dose conversion coefficients for use in external radiation protection.

## Conclusion

The IAEA report recommended establishing a reference phantom specific for various regions in Asia for internal dose evaluation and radiation protection. To address this need, this study constructed Taiwanese reference phantoms based on the average height, weight, and organ mass of Taiwanese adults. The SAF and S-value derived from TRM and TRW were compared to those derived from the ORNL adult phantom, ORNL female phantom, Japanese reference phantom, and Korean reference phantom. The SAF and S-value of TRM and TRW are markedly different from those of the ORNL phantoms, and the disparity between phantoms can lead to errors up to 8 times in internal dose evaluation. The developed anthropomorphic phantoms are expected to be employed for internal exposure to increase the accuracy of dose evaluation for Taiwanese.
